# Impact of a Patient Support Program on time to discontinuation of adalimumab in Australian adult patients with immune-mediated inflammatory diseases–an observational study

**DOI:** 10.1371/journal.pone.0300624

**Published:** 2024-06-13

**Authors:** Graeme Jones, Miriam Calao, Jakob Begun, Shirley Sin, Mahsa H. Kouhkamari, Elisa Young, Pablo Fernández-Peñas, Alan Watts, Andrew J. Östör

**Affiliations:** 1 Menzies Institute for Medical Research, University of Tasmania, Hobart, Tasmania, Australia; 2 Abbvie Pty Ltd, Mascot, New South Wales, Australia; 3 Mater Research, Brisbane, QLD, Australia; 4 Department of Gastroenterology, Mater Hospital, Brisbane, QLD, Australia; 5 Prospection Pty Ltd, Sydney, NSW, Australia; 6 Southern Star Research Pty Ltd, Gordon, Australia; 7 Department of Dermatology, The University of Sydney, Westmead Hospital, Sydney, Australia; 8 Melbourne and ANU, Canberra & Emeritus Research, Monash University, Melbourne, Australia; Osaka University of Pharmaceutical Sciences, JAPAN

## Abstract

This observational study evaluated the impact of a sponsor company-provided Patient Support Program (PSP) on discontinuation of adalimumab in adult Australian patients eligible for Pharmaceutical Benefit Scheme (PBS)-reimbursed adalimumab for Rheumatoid Arthritis (RA), Ankylosing Spondylitis (AS), Psoriatic Arthritis (PsA), Crohn’s Disease (CD), Ulcerative Colitis (UC), or Hidradenitis Suppurativa (HS). Patients initiating adalimumab between May 2018 and September 2019 were enrolled into two prospective cohorts based on their decision to opt for or decline the PSP (PSP or non-PSP cohorts). In addition, a historical, retrospective Non-PSP cohort was established from the Services Australia 10% PBS dataset by extracting data of patients initiating adalimumab prior to the introduction of adalimumab PSPs and based on adalimumab PBS listing dates (AS: April 2007 to March 2009; PsA/RA: January 2007 to December 2008; CD: January 2009 to December 2010; HS and UC indications not included). Follow-up for all cohorts was 12 months. The primary endpoint was the time to discontinuation, compared between the prospective PSP cohort and the prospective or retrospective Non-PSP cohort. Inverse probability of treatment weighting was used to balance the cohorts. A Cox proportional hazards model indicated no difference in time to discontinuation between the prospective PSP (n = 162) and non-PSP (n = 65) cohorts (HR [95% CI] = 1.256 [0.616–2.563], p = 0.5304). The 12-month adalimumab persistence rates (95% CI) were 78% (69%, 84%) and 82% (67%, 90%), respectively. In contrast, discontinuation was less likely in the prospective PSP (n = 151) compared with the retrospective non-PSP (n = 297) cohort (HR [95% CI] = 0.44 [0.28–0.68], p<0.001). The 12-month persistence rates (95% CI) were 81% (76%, 90%) and 61% (56%, 67%), respectively. Overall, this study suggests that optimal adalimumab persistence can be achieved with either a structured PSP or healthcare support from other sources, but this was not the case more than a decade ago.

## Introduction

Adalimumab is a human monoclonal anti-tumour necrosis factor (TNF) antibody [1], approved for use in Australia as a biological disease-modifying anti-rheumatic drug (bDMARD) for the treatment of immune-mediated inflammatory diseases (IMIDs) including rheumatoid arthritis (RA), ankylosing spondylitis (AS), psoriatic arthritis (PsA), Crohn’s disease (CD), ulcerative colitis (UC), and hidradenitis suppurativa (HS) [2]. These diseases are characterized by acute or chronic inflammation that can affect any organ system [3] and have significant impact on patients’ health and quality of life [4, 5]. Rheumatoid arthritis is the most prevalent of these conditions, affecting an estimated 1.9% of Australians [6] and ranking 14th among the leading causes of disease burden in Australia in 2022 [7]. Despite their proven efficacy [8], high rates of bDMARD discontinuation can be observed in real world settings [9, 10]. In Australia, bDMARDs are predominantly accessed through the Pharmaceutical Benefits Scheme (PBS) for patients who meet disease severity criteria prior to bDMARD initiation, and who achieve specified response criteria over time to remain eligible for ongoing reimbursement and supply. If patients do not respond satisfactorily to initial bDMARD therapy, or have adverse events leading to discontinuation of therapy, clinicians are able to switch patients (if clinically appropriate) onto alternative treatments. Clinicians are also able to apply for reimbursement approval changes for reasons apart from efficacy and/or safety. Whilst the most commonly reported reasons for treatment discontinuation are lack of efficacy and adverse events [11–14], other factors may also impact bDMARD discontinuation, such as the reimbursement process (including co-administration of therapeutically complementary DMARDs, speed to treatment initiation, and meeting requirements for ongoing reimbursement eligibility), or patient/clinician preferences for alternative product characteristics (for example different frequency or route of administration). Poor persistence with and nonadherence to these therapies can undermine their effectiveness [15], and treatment response is often more likely to decline with each subsequent line of bDMARD therapy compared with bDMARD-naïve patients [13, 14, 16–18]. Hence, interventions supporting optimal bDMARD use to improve persistence and adherence in patients who require long-term treatment, may improve patients’ outcomes.

Patient education and support in the form of Patient Support Programs (PSPs) are postulated to positively influence bDMARD persistence, and hence patients’ outcomes, through optimisation of factors including correct timing of doses, reduced missed doses, optimal self-injection technique, lifestyle education and support, and education on the medicine.

However, there is mixed evidence regarding the benefits of PSPs. There is evidence from a broad range of therapeutic areas that patient utilisation of PSPs improves medication adherence and persistence [19–22], although studies reporting no significant benefit have also been published [23]. This may suggest that the potential benefit of PSPs may be influenced by factors such as medical condition, drug class, dosing regimen, PSP specific offerings and degree of their uptake by patients, PSP services format and accessibility, as well as the level of independent support provided to patients by their treating physician/practice.

In support of the quality use of medicines, AbbVie (the Australian company, AbbVie Pty Ltd) has offered a PSP for Australian patients prescribed HUMIRA® (adalimumab) since 2009, which has evolved over time. The PSP includes several offerings and services that enrolled patients can opt to uptake depending on their needs.

The primary objective of this study was to estimate the potential incremental benefit of the HUMIRA® (adalimumab) PSP, as measured by its impact on the time to discontinuation after first-time adalimumab initiation in Australian adult (≥18 years of age) patients eligible for PBS-reimbursed adalimumab. Three cohort of patients were established. Patients prescribed adalimumab by their clinician between May 2018 and September 2019 were enrolled in a prospective PSP or non-PSP cohort based on their decision to opt for or decline the PSP. In addition, a historical, retrospective cohort comprising patients who initiated adalimumab prior to the implementation of the PSP in 2009 from a sample of claim records from the Services Australia 10% Pharmaceuticals Benefits Scheme (PBS) dataset (PBS 10% sample) [24] was also established. These patients can be considered a less biased non-PSP cohort as they had no option to enrol onto a PSP even when no alternative support was available to them. In addition, the retrospective cohort also provides an opportunity to assess the impact of changes in standard of care over time for patients not enrolled in a PSP. The primary endpoint, time-to-discontinuation in the 12-month period after initiation of adalimumab, was designed to test the null hypothesis that there is no difference in the time to discontinuation of adalimumab between the prospective PSP cohort compared with the prospective non-PSP cohort or compared with the retrospective non-PSP cohort. The impact of PSP enrolment on rates of adalimumab persistence up to 12 months after initiation, and on patient-reported outcome measures (PROMs) in the prospective cohorts, were also evaluated as secondary objectives.

## Methods

### Ethical considerations

This observational study was approved by two Human Research Ethics Committees (St Vincent’s Hospital Melbourne Human Research Ethics and Bellberry Human Research Ethics Committee). Permission for use of the non-PSP PBS 10% sample was sought from Services Australia’s External Request Evaluation Committee (EREC MI6714). Patients prospectively enrolled were provided with a written Informed Consent Form (ICF) containing adequate information to allow them to make an informed decision to participate in the study and were given ample opportunity to discuss the study with site staff and have any questions adequately answered. The ICF was signed before any study procedures were performed. Authors who were also Principal Investigators for the study may have had access to only their own patients’ data during data collection, however for the manuscript development all authors were only provided de-identified and/or aggregated data.

### Study design and endpoints

This was an observational cohort study with the primary objective of estimating the potential effect of the PSP on HUMIRA® (adalimumab) persistence in adult (≥ 18 years of age) Australian patients initiating for the first time adalimumab, as defined by the time to discontinuation. The primary endpoint was to compare the time to discontinuation between a prospective cohort of PSP patients, and either a prospective non-PSP cohort or a retrospective non-PSP cohort established from the PBS 10% sample.

The secondary objectives were to estimate the potential impact of the PSP on patient-reported outcomes measures (PROMs), including: health-related quality of life using the EQ-5D-5L (rheumatology and gastroenterology indications) or Dermatology Life Quality Index (DLQI; HS only); attitudes to medicines in general using the Beliefs about Medicines Questionnaire (BMQ):General (all indications); effect of health problems on ability to work and perform regular activities using the Work Productivity and Activity Impairment (WPAI)-General Health (GH) V2.0 (all indications); global assessment using the Patient Global Assessment of Disease Activity (PGDA; RA and PsA); evaluation of disease activity using the Bath Ankylosing Spondylitis Disease Activity Index (BASDAI; AS only). The DLQI was used as an alternative to the EQ-5D-5L for HS, as the EQ-5D-5L is not considered a validated tool with adequate disease activity discrimination for dermatological conditions. The PGDA was used for RA and PsA only since this measure has been most widely used in these patient groups. The secondary endpoints included change from baseline in PROMs scores, and the proportion of patients with a reduction in PGDA scores of 25% and 50% from baseline, up to 12 months after initiation of adalimumab in the prospective PSP versus non-PSP cohort.

Additional secondary endpoints included assessing the impact of PSP enrolment on rates of adalimumab persistence up to 12 months after initiation and analyses by disease indications or pooled indications where the sample size allowed it, with the aim of describing any potential differences by specific disease or therapeutic area.

Supplementary analyses included time to adalimumab discontinuation compared between the prospectively enrolled patients who reported having received self-injection training and those who reported no self-injection technique training.

### Subjects–Prospective cohorts

Subjects were eligible to be enrolled in the prospective cohorts of this study if they: were Australian adult (≥ 18 years of age); had a diagnosis of RA, AS, PsA, CD (moderate to severe refractory CD or fistulizing CD), UC, or HS by their treating specialist physician; were prescribed PBS-reimbursed adalimumab for the first time (initiation course) for their diagnosed condition; had not been administered the first dose at the time of study enrolment.

Patients decided whether to enrol in the PSP after a joint clinical/patient decision had been made to initiate adalimumab, and prior to being approached for potential recruitment to the study. Following screening and informed consent, patients who had opted to enrol in the PSP were included in the study’s prospective PSP cohort, while those who did not were included in the prospective non-PSP cohort. Enrolled patients were prospectively recruited from 12 urban Australian sites (7 public and 5 private), comprising specialist rheumatology (5 sites), gastroenterology (3 sites) and dermatology (4 sites) sites between May 2018 to September 2019. Enrolled patients were followed-up for a maximum of 12 months after administration of the first dose of adalimumab. Recruitment processes included study advertisement emails sent to patients enrolled in the PSP, printed study brochures/flyers provided to potentially eligible patients through HCPs at specialist practices (rheumatologists, gastroenterologists, and dermatologists) or at investigative sites, and a study registration of interest website.

Following enrolment and baseline visit procedures, patients were asked to complete baseline PROMs prior to their first dose of adalimumab. Additional PROMs were completed via an online database at timepoints according to indication.

### Subjects–Retrospective cohort

A retrospective cohort comprising Australian adult (aged ≥ 18 years) patients who initiated adalimumab prior to the implementation of the PSP in 2009 was also established as a non-PSP control cohort and consisted of historical patient data extracted from the Australian Department of Human Services PBS 10% sample data, which is a nationally representative longitudinal systematic random sample of 10% of the Medicare-eligible Australian population and all of their reimbursed dispensations [24–27].

Patients were included in the retrospective non-PSP cohort if they had been dispensed an initial prescription for adalimumab (initiation course) during the sample selection window for a diagnosis of RA, PsA, or CD, based on PBS item codes. UC and HS indications were not included in the retrospective analyses as adalimumab was not approved and reimbursed by the PBS for these indications prior to the introduction of the first adalimumab PSP. Patients must not have previously used adalimumab and patients with multiple diagnoses were excluded.

Data sampling windows (including 12 months adalimumab sample selection window followed by up to 12 months follow up) were: April 2007 until March 2009 for AS; January 2007 until December 2008 for PsA and RA, and January 2009 until December 2010 for CD (including refractory CD only as fistulising CD was not PBS reimbursed until April 2011). AbbVie PSP program data showed that there were only 6 Crohn’s disease patients enrolled in the PSP during 2009, indicating that the 2009–2010 PBS 10% sample dataset represents a valid ‘non-PSP’ like dataset for this indication.

Data were extracted and analysed by Prospection Pty Ltd, a health analytics company approved to undertake analytics on data extracted from the Services Australia 10% PBS dataset under a licence agreement with Services Australia. This study and publication of subsequent results were approved by the Australian Government Department of Human Services External Request Evaluation Committee and complied with relevant data protection and privacy regulations.

### Patient support program

The Australian HUMIRA® PSP is sponsored by AbbVie (the Australian company, AbbVie Pty Ltd) and is available to Australian patients prescribed HUMIRA® (adalimumab). It was first launched in 2009 and offerings of the program have changed over time. At the time of this study, the PSP included a range of general components such as a Welcome kit (sharps bin, patient booklet, cold pack, step by step injection guides, QR codes to injection videos, fridge magnet injection reminder); and access to a website where patients can learn about HUMIRA® (adalimumab), disease state and the PBS, access safety information, request a nurse injection training session, set up reminders (treatment, appointments and prescription collection), learn how to travel with HUMIRA® (adalimumab), order replacement sharps bin, order travel solutions (containers to maintain product temperature whilst travelling), request a health coach (dietician/nutritionist or exercise physiologist), information on mental health resources, sharps disposal information, patient organisations information and additional resources. Patients enrolled into the PSP decide which of the services available they want to use.

### Variables–Prospective cohorts

In the two prospective cohorts, discontinuation was defined as the interruption of continual adalimumab therapy for at least 3 months between doses. If the patient was switched onto an alternate bDMARD, then discontinuation was confirmed without the need for further follow-up. Periods of less than 3 months between doses were defined as temporary dose delays, not meeting the criteria for discontinuation. The 3-month interruption interval defining discontinuation was chosen as it represented the minimum period when it was likely that a patient could be determined to have discontinued, taking into account that patients ordinarily have to pick up their scripts monthly from the pharmacy and their treating physician would normally review their need for scripts every 3 to 6 months. This period would also allow for potential delays in picking up scripts or temporary interruptions. If a potential adalimumab discontinuation occurred between 9 and 12 months after the first dose, patients were followed up by a telephone call from the investigative site to confirm whether the patient eventually met the definition of adalimumab discontinuation. bDMARD line of therapy was derived from the CRF (Case Report Form) page where patients were asked to select prior b/tsDMARDs (biologic/targeted synthetic DMARDs) received. If the patients did not select prior b/tsDMARD use, they were categorised as line 1, and if patients did indicate other b/tsDMARD(s) prior to adalimumab, they were categorised as line 2+. Additional source data included information on adalimumab dose escalations and co-prescribed medications entered directly by patients into the Patient Medication Diary via the ePRO database throughout follow-up. The main reason for discontinuation was determined by the patient’s treating clinician and entered into the CRF by study site staff. The clinician reported discontinuation options in the CRF included: due to adverse event, due to lack of therapeutic efficacy, due to loss of efficacy, due to process failure, due to patient/HCP/payer preference decisions unrelated to efficacy or adverse events, lost to follow up. Patient-reported reasons for discontinuation were collected, but were not used for sub-analyses, rather to assist investigators to seek confirmation of the main reason for any adalimumab discontinuations. Where the date of first injection was missing, or partial, for a patient, and there was post-baseline data to indicate that the patient did start adalimumab, the baseline assessment date was imputed as adalimumab start date (the latest date was imputed when assessments were completed over a number of days). Time to drug discontinuation for adalimumab was calculated based on the start and stop dates that were patient-reported in the Patient Medication Diary. The CRF also recorded whether patients had received training from specialised nurses for self-injection technique for adalimumab, based on patient self-report at the 3-month timepoint. PROMs were entered into an electronic Patient-Reported Outcomes (ePRO) database. Within the database there were no restricted windows for completion of the baseline questionnaires; if any baseline data were entered by a patient in the ePRO the day after first adalimumab dose or later, the patient remained in the study on the rationale that the patient would be expected to have less severe disease intensity measures at the delayed baseline than if baseline PROMs were measured prior to the first dose. Any impact on endpoints would therefore be expected to be conservative and potentially reduce the estimate of PSP impact. Data from questionnaires to be completed at weekly intervals were collected within 3 days of the expected completion date of the questionnaire. Data from questionnaires to be completed at monthly or 3 monthly intervals were collected within two weeks of the expected completion date of the questionnaire.

### Variables–Retrospective non-PSP PBS 10% cohort

In the retrospective non-PSP PBS 10% cohort, discontinuation was defined as the date of the last prescription for adalimumab plus the length of time the filled prescription is known to last (28 days), where there is no subsequent prescription of adalimumab in the following 3-month period. Subjects without an observed discontinuation event were censored at the end of the study window. Gender was available in the dataset. Age was calculated based on date of birth and index date for each patient. Line of therapy was defined based on prior use of bDMARDs. If patients had no dispensing for bDMARDs prior to adalimumab, they were categorised as line 1 and if patients had a dispensing for another bDMARD prior to adalimumab, they were categorised as line 2+. Concomitant treatments with cDMARD (conventional DMARD) or corticosteroids were analysed 3, 6 and 12 month post index date.

### Statistical methods–Sample size

The sample size calculation was based on the cross-indication analysis of the primary endpoint (12-month adalimumab time to discontinuation) with significance set at 0.05 and the hypothesis test 2-sided. The study aimed to recruit 420 patients (PSP + non-PSP cohorts) initiated on adalimumab for the first time after meeting PBS-reimbursement criteria, in an approximate 2:1 ratio between the PSP and non-PSP cohorts (280:140), estimated to provide a power of 81% with 165 discontinuations and 83% with 180 drug discontinuations, respectively, to detect a hazard ratio of 0.645. The actual sample size of the prospective cohorts was lower than planned (Full analysis Set [FAS]: N = 170 PSP cohort, N = 75 non-PSP cohort). The retrospective non-PSP cohort FAS was N = 297.

### Statistical methods–Prospective comparisons

The general analytical approach for all endpoints was descriptive in nature. All descriptive and outcome data was summarised and analysed by PSP status and Total (PSP and prospective Non-PSP combined), with indication subgrouping when appropriate, based on sample size in each category. Pooled indications were defined as Rheumatology (RA, PsA and AS) and Gastroenterology (CD and UC). Two datasets were established. The FAS consisted of all patients who were recruited into the study, who had a valid adalimumab start date and/or at least one post-baseline follow-up for continuation of adalimumab. Where the date of first injection was missing, or partial, for a patient, and there was post-baseline data to indicate that the patient did start adalimumab, the latest baseline assessment date was imputed as the adalimumab start date. Within the FAS, grouping into PSP/non-PSP was based on the PSP enrolment intention recorded at baseline. The “Primary Analysis Set” consisted of all FAS patients who had valid values for all covariates included in the propensity score model used to weight the data within the endpoint analyses.

Survival analyses were performed to assess the primary endpoint, with an ‘event’ defined as a drug discontinuation. Time to adalimumab discontinuation was compared between the PSP and prospective non-PSP cohort by using an inverse probability of treatment weighting (IPTW) approach. A propensity model (logistic regression) was fitted for all patients included in the analysis using the PROC PSMATCH procedure to estimate the propensity score (PS) of participating in the PSP for each patient, incorporating the following baseline covariates: age, sex, disease duration (derived from year of clinical diagnosis, round up to the nearest year), disease indication, health-related quality of life at adalimumab initiation, concomitant treatment with non-biological DMARDs (Yes|No), highest education level (graduate or above|other). bDMARD line of therapy was not included in the final propensity model to optimize number of patients with no missing data that could be included in the model, given that the propensity score models including this variable were found to make negligible contribution to the overall model and subsequent analyses. Based on the estimated PS, stabilised IPTW, the inverse probability of participating in the PSP (PSP or non-PSP), was estimated for each patient by including the PSWEIGHT statement in the PSMATCH procedure. A weighted Cox regression, weighted by the stabilised IPTW, was used to compare the time to adalimumab discontinuation between PSP and non-PSP. The Cox model estimated the hazard-ratio (HR) comparing the hazard (risk) of drug discontinuation in the PSP cohort to the hazard (risk) in the non-PSP cohort. A sensitivity analysis was undertaken using propensity score trimming, where stabilised IPTWs below the 5th and above the 95th percentile were excluded. A second sensitivity analysis was undertaken whereby the weighting was omitted from the Cox regression, allowing the analysis to include all patients in the FAS. Changes from baseline for PROM scores were analysed over time with a mixed model for repeated measurements (MMRM). MMRM model was also weighted by IPTW. Time to adalimumab discontinuation was also compared between the prospectively enrolled patients who reported having received self-injection training and those who reported no self-Injection training, using an unweighted Cox Regression. The Cox Regression model was implemented to calculate the adjusted hazard-ratio (HR), to compare the hazard (risk) of drug discontinuation in the Self-Injection Training cohort to the hazard (risk) in the No Self-Injection Training cohort. All prospective cohort analyses were performed using SAS® Version 9.4 (SAS Institute, Cary, North Carolina, USA).

### Statistical methods–Prospective PSP versus retrospective non-PSP analyses

A similar IPTW approach, as applied for the prospective analyses, was also used for the comparisons against the retrospective PBS 10% non-PSP cohort. The independent covariates used as predictor variables in the propensity score model for these analyses were (based on available covariates in the PBS 10% dataset): age, sex, concurrent treatment, bDMARD line of treatment. The FAS comprised the full analysis set for the PSP prospective cohort for RA, AS, PsA and CD, and all available data from the PBS 10% dataset within the specified sampling window and meeting the inclusion criteria. Comparisons between the PSP and non-PSP PBS 10% cohort were conducted using a “Primary Analysis Set”, including all FAS patients who had valid values for all covariates included in the propensity score model. Two sensitivity analyses were performed, the first one using a trimmed analysis set similarly to the prospective cohorts’ analyses, and one using a dataset which included an additional year of follow-up time for the non-PSP PBS 10% sample. This dataset allows the exploration of temporal patterns of drug discontinuation and drug persistence. This sensitivity analysis was deemed not to compromise the non-PSP definition of the cohort, as it was likely that there was less than 10% PSP ‘contamination’ based on PSP enrolment data during those years. All analyses involving the retrospective cohort were performed using Prospection’s PharmDash software or R, as appropriate.

## Results

### Participants

**Fig 1:**
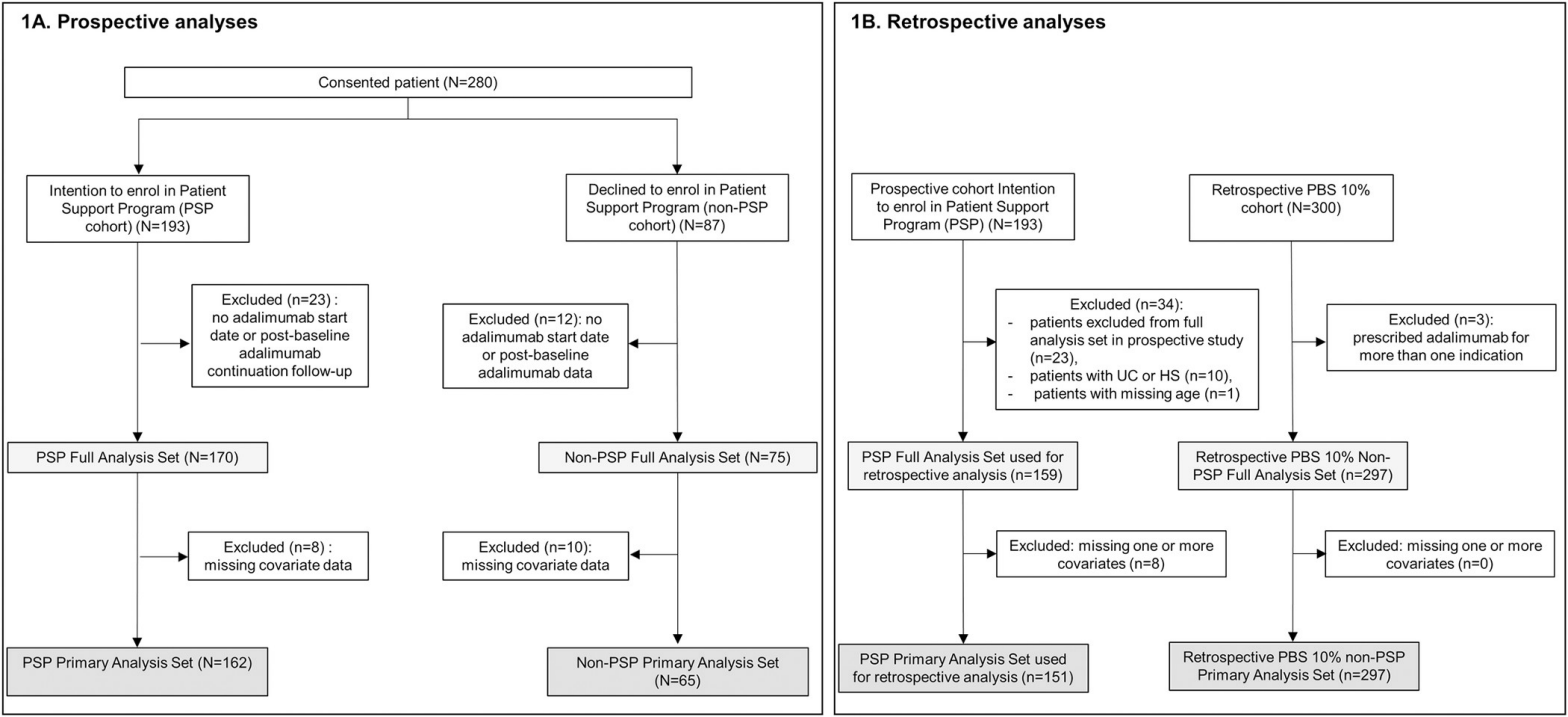
Patient flow. Patient flow for prospective PSP versus prospective non-PSP analyses (1A) or prospective PSP versus retrospective non-PSP analyses (1B).

Patient flow is summarized in Fig 1.

A total of 280 patients were recruited across the two prospective cohorts (193 in the PSP cohort and 87 in the non-PSP cohort) over 17 months from May 2018 to September 2019 and were included in the FAS for the prospective analyses (Fig 1A). A total of 159 patients from the prospective PSP and 297 from the non-PSP PBS 10% sample were included in the FAS for the analyses against the retrospective cohort (Fig 1B). The Primary Analysis Set used for the final weighted and adjusted model included 162 PSP patients and 65 Non-PSP patients for the prospective analyses (1A), while it included 151 patients in the prospective PSP cohort and 297 in the retrospective PBS 10% non-PSP cohort for the analyses against the retrospective cohort (Fig 1B). Unless differently specified, only the outcomes from the Primary Analysis Set are described here.

Most of the patients in any cohort were prescribed adalimumab for a rheumatology condition (172/227 [76%] total rheumatology patients for prospective analysis; 336/448 [75%] total rheumatology patients for retrospective analysis). Within rheumatology indications, most of the patients had a diagnosis of RA (Table 1).

**Table 1:** Patient disposition, demographics and baseline characteristics (Primary Analysis Set).

**1A. Primary Analysis Set for prospective PSP vs prospective non-PSP analyses**				
** **	** **	Cross-indication*		
** **	** **	**PSP (N = 162)**	**non-PSP Prospective (N = 65)**	**Total (N = 227)**
**Primary condition for which the patient is receiving adalimumab treatment**	Ankylosing spondylitis	21	9	30
	Crohn’s Disease	34	7	41
	Hidradenitis Suppurativa	7	4	11
	Psoriatic arthritis	37	19	56
	Rheumatoid arthritis	60	26	86
	Ulcerative Colitis	3	0	3
Age—mean years (SD)**		49 (14)	51 (15)	50 (14)
**Female n (%)**		107 (66)	42 (65)	149 (66)
**Ethnicity n (%)**	Caucasian	157 (97)	60 (92)	217 (96)
	Asian	1 (1)	3 (5)	4 (2)
	Other	4 (2)	2 (3)	6 (3)
**Born in Australia n (%)**		133 (82)	49 (75)	182 (80)
**Aboriginal or Torres Strait Island origin n (%)**	Aboriginal	1 (1)	1 (2)	2 (1)
**Smoking status n (%)**	never	83 (51)	30 (46)	113 (50)
	ex-smoker	64 (40)	29 (45)	93 (41)
	current	15 (9)	6 (9)	21 (9)
**Highest education level—binary n (%)**	Primary/High school or TAFE / Technical College	77 (48)	38 (58)	115 (50)
	University—Undergraduate or Postgraduate	85 (52)	27 (42)	112 (49)
**Adalimumab line of therapy—binary n (%)** ^**¶**^	First	144 (91)	53 (84)	197 (89)
	Second or more	14 (9)	10 (16)	24 (11)
**Self-injection trainings received n (%)** ^**¥**^	Yes	91 (71)	24 (49)	115 (64)
	No	38 (29)	27 (53)	65 (36)
**Concurrent immunomodulators n (%)**		92 (57)	38 (58)	130 (57)
**Weight (Kg) at baseline–mean (SD)** ^**†**^		84 (21)	81 (18)	83 (20)
**1B. Primary Analysis Set for prospective PSP vs retrospective non-PSP analyses**				
** **	** **	Cross-indication*		
		**PSP (N = 151)**	**non-PSP PBS 10% (N = 297)**	**Total (N = 448)**
**Primary condition for which the patient is receiving adalimumab treatment**	Ankylosing spondylitis	21	53	74
	Crohn’s Disease	32	80	112
	Psoriatic arthritis	37	42	79
	Rheumatoid arthritis	61	122	183
Age -mean years (SD)***	Mean (SD)	50 (14)	48 (14)	49 (14)
**Female n (%)**		102 (68)	170 (57)	272 (61)

**Adalimumab line of therapy n (%)**	first	137 (91)	221 (74)	358 (80)
	second or more	14 (9)	76 (26)	90 (20)
**Concurrent immunomodulators n (%)**		110 (73)	176 (59)	286 (64)

* Pooled Rheumatoid Arthritis (RA), Ankylosing Spondylitis (AS), Psoriatic Arthritis (PsA), Crohn’s Disease (CD), Ulcerative Colitis (UC), and Hidradenitis Suppurativa (HS)

** Age calculated at date of informed consent

^**¶**^ Percentages based on the number of patients with non-missing data (PSP n = 158; non-PSP n = 63): total number of patients with missing data: n = 6.

^**¥**^ Percentages based on the number of patients with non-missing data (PSP n = 129; non-PSP n = 51): total number of patients with missing data: n = 47.

^**†**^ Based on the number of patients with non-missing data (PSP n = 161; non-PSP n = 64): total number of patients with missing data: n = 2.

*** Age calculated from cohort in PSP group or as recorded in non-PSP PBS 10%percentages were calculated based on the number of patients in the group

Across all indications, the distribution of patient characteristics was similar in the PSP and Non-PSP groups, except for slightly numerically lower percentages in the non-PSP prospective group of patients born in Australia (PSP: 82%; non-PSP: 75%) and having obtained a university degree (PSP: 52%; non-PSP 42%) compared to the PSP group, and a lower numerical percentage in the non-PSP PBS 10% of first line adalimumab users (PSP: 91%; non-PSP PBS 10%: 74%) compared to the PSP group (Table 1). Between 57% and 68% of the patients were female in the cohorts (Table 1). Overall, the majority of patients were first line bDMARD users (91% of patients in the PSP prospective cohorts used in the prospective and retrospective analyses; 84% in the prospective non-PSP cohort; and 74% in the non-PSP PBS 10% cohort) (Table 1). Similar trends were observed in the Rheumatology pooled patients, while within the prospective RA subgroup, the proportion of patients treated with adalimumab as a first-line bDMARD therapy was notably lower in the Non-PSP group (77%) compared with the prospective cross-indication Non-PSP group (84%) and the prospective PSP group (92%).

Self-injection training status (confirmation of trained or untrained) was reported by 180 patients across all indications (129 [80%] PSP patients and 51 [78%] Non-PSP patients) (Table 1A). Across all indications, the proportion of patients who reported having received self-injection training was higher in the PSP group (91 [71%] patients) than the Non-PSP group (24 [47%] patients) (Table 1A).

### Time to discontinuation

**Fig 2:**
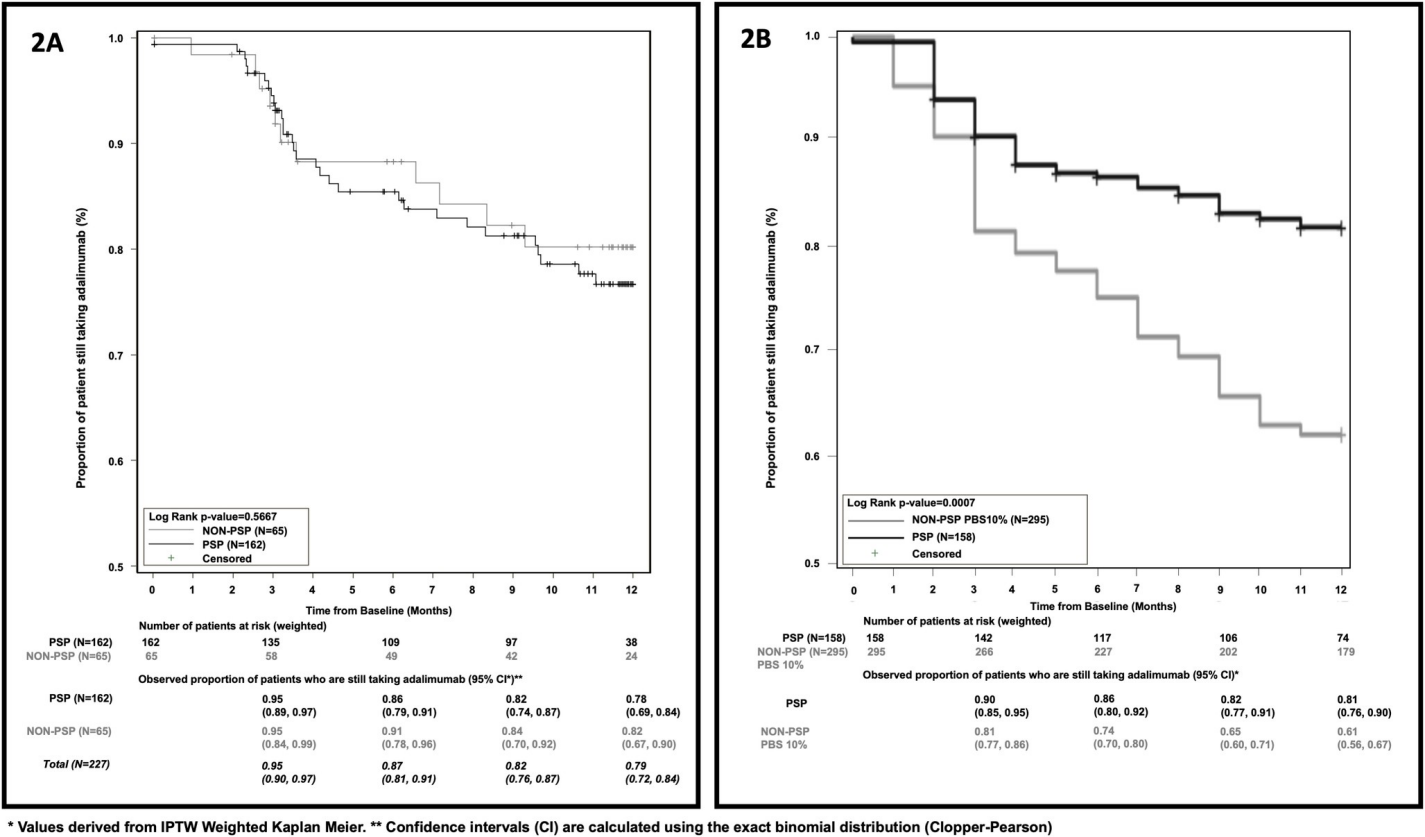
Adalimumab time to discontinuation Kaplan–Meier curves and 12-month persistence rates across all indications. Primary Analysis Sets, analyses adjusted by the inverse probability of treatment weighting (IPTW). The log rank score P value for the persistence survival curve shown. 2A) Prospective PSP cohort versus prospective non-PSP cohort. 2B) Prospective PSP cohort versus retrospective PBS 10% non-PSP cohort.

Kaplan-Meir curves for adalimumab time to discontinuation and associated log rank score p value are presented in Fig 2.

**Fig 3:**
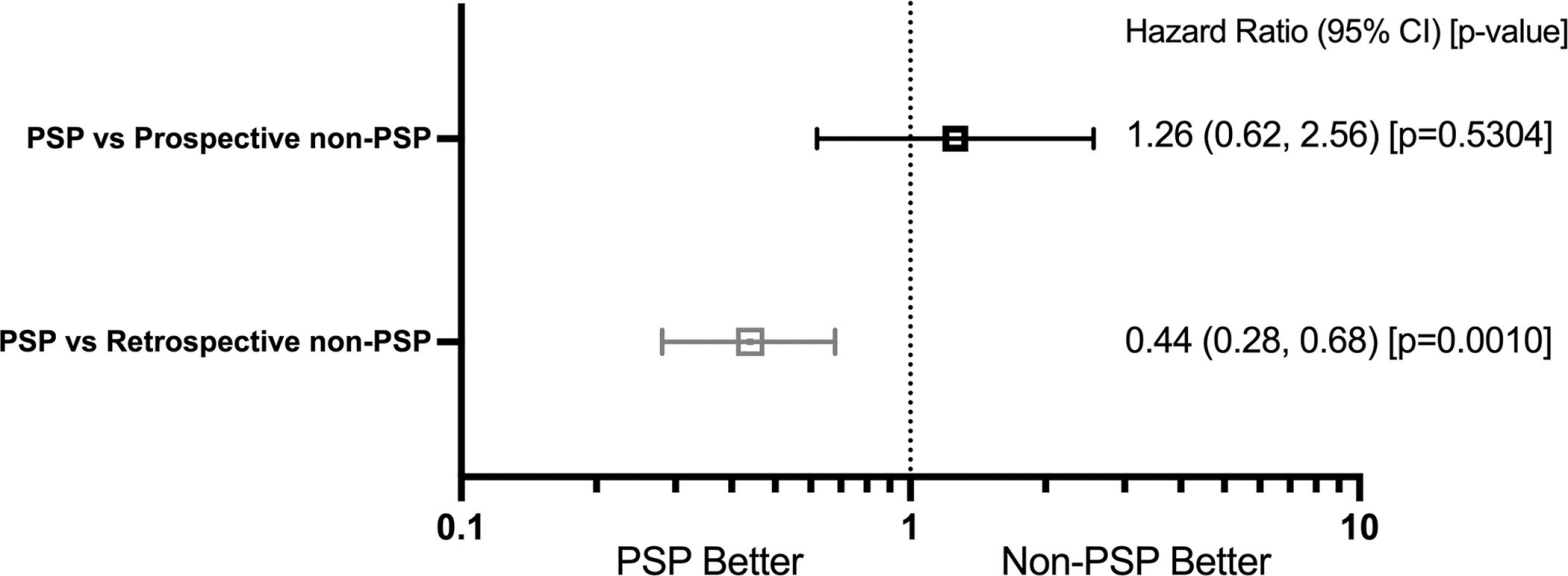
Hazard-Ratio (HR) for adalimumab discontinuation across all pooled indications. A Cox regression, weighted by the inverse probability of treatment weighting (IPTW), was used to calculate the hazard-ratio (HR) comparing the hazard (risk) of drug discontinuation in the PSP cohort to the hazard (risk) in the non-PSP cohorts (prospective and retrospective) across all indications.

In the final weighted and adjusted model for the comparison between the two prospective PSP and non-PSP cohorts, participation in the PSP was not significantly associated with adalimumab discontinuation, as determined by log-rank test (Fig 2A) and Cox Regression (Fig 3). Weighted Kaplan-Meir estimates of adalimumab 12-month persistence rates were 0.79 (95% CI 0.72, 0.84) in the overall prospective population, 0.78 (95% CI: 0.69, 0.84) in the prospective PSP cohort and 0.82 (95% CI: 0.67, 0.90) in the prospective non-PSP cohort (Fig 2A).

S1 Fig presents time to discontinuation Kaplan Meier curves in the RA and Rheumatology prospective subgroups. Both subgroup analyses demonstrated no significant difference in the survival functions of adalimumab time to discontinuation between the PSP and Non-PSP prospective cohorts. The hazard ratio findings are also presented in S3 Fig. The sample size was deemed insufficient to run indication-specific analyses for AS, PsA, CD, UC and HS or pooled Gastroenterology indications. A sensitivity analysis was also undertaken using propensity score trimming whereby stabilised IPTWs below the 5th and above the 95th percentile were excluded, and the weighted Cox regression was repeated using the trimmed population. The results, presented in S1 Table, were similar to the non-trimmed analysis. A second sensitivity analysis was undertaken whereby the weighting was omitted from the Cox regression, allowing the analysis to include all patients in the FAS. The results of this sensitivity analysis, presented in S1 Table, were also consistent with the main analysis.

As shown in Table 2, overall the most common clinician-reported reason for discontinuation across all indications was “Lack of therapeutic efficacy” (28 [51%] patients), followed equally by “Loss of Efficacy” (11 [20%] patients) and “Adverse Event” (11 [20%] patients). Adverse events were reported being a reason for discontinuation in 27% of Non-PSP patients (n = 4) and 17.5% of PSP patients (n = 7). Discontinuations for reasons unrelated to efficacy or adverse events were only reported by 2 (3.5%) discontinuing patients overall. Reasons for discontinuation in the RA and Rheumatology subgroups were similar to the cross-indication group, with “Lack of therapeutic efficacy” most frequently reported (Rheumatology: 27 [54.0%] patients; RA: 12 [50.0%] patients) followed by “Loss of Efficacy” (Rheumatology: 11 [22.0%] patients; RA: 5 [20.8%] patients) and “Adverse Event” (Rheumatology: 9 (18.0%) patients; RA: 5 [20%] patients).

**Table 2:** Primary reason for discontinuation (clinician reported) in the prospective cohorts (Primary Analysis Set).

	Cross-indication*		
n (%)	PSP (N = 162)	non-PSP Prospective (N = 65)	Total (N = 227)
Number of patients who discontinued adalimumab	42 (26)	15 (23)	57 (25)
Due to adverse event**	7 (17.5)	4 (27)	11 (20)
Due to lack of therapeutic efficacy**	19 (47.5)	9 (60)	28 (51)
Due to loss of efficacy**	9 (22.5)	2 (13)	11 (20)
Due to patient/HCP/payer preference decisions unrelated to efficacy or adverse events**	1 (2.5)	0	1 (2%)
Due to process failure**	1 (2.5)	0	1 (2)
Reason for discontinuation unable to be clinically confirmed**	3 (7.5)	0	3 (5)

* Pooled Rheumatoid Arthritis (RA), Ankylosing Spondylitis (AS), Psoriatic Arthritis (PsA), Crohn’s Disease (CD), Ulcerative Colitis (UC), and Hidradenitis Suppurativa (HS)

** Percentages based on the number of patients who discontinued adalimumab (based on reporting the end date of adalimumab and/or reporting the reason for discontinuation [clinician-reported]) with non-missing data for the given parameter

**Fig 4:**
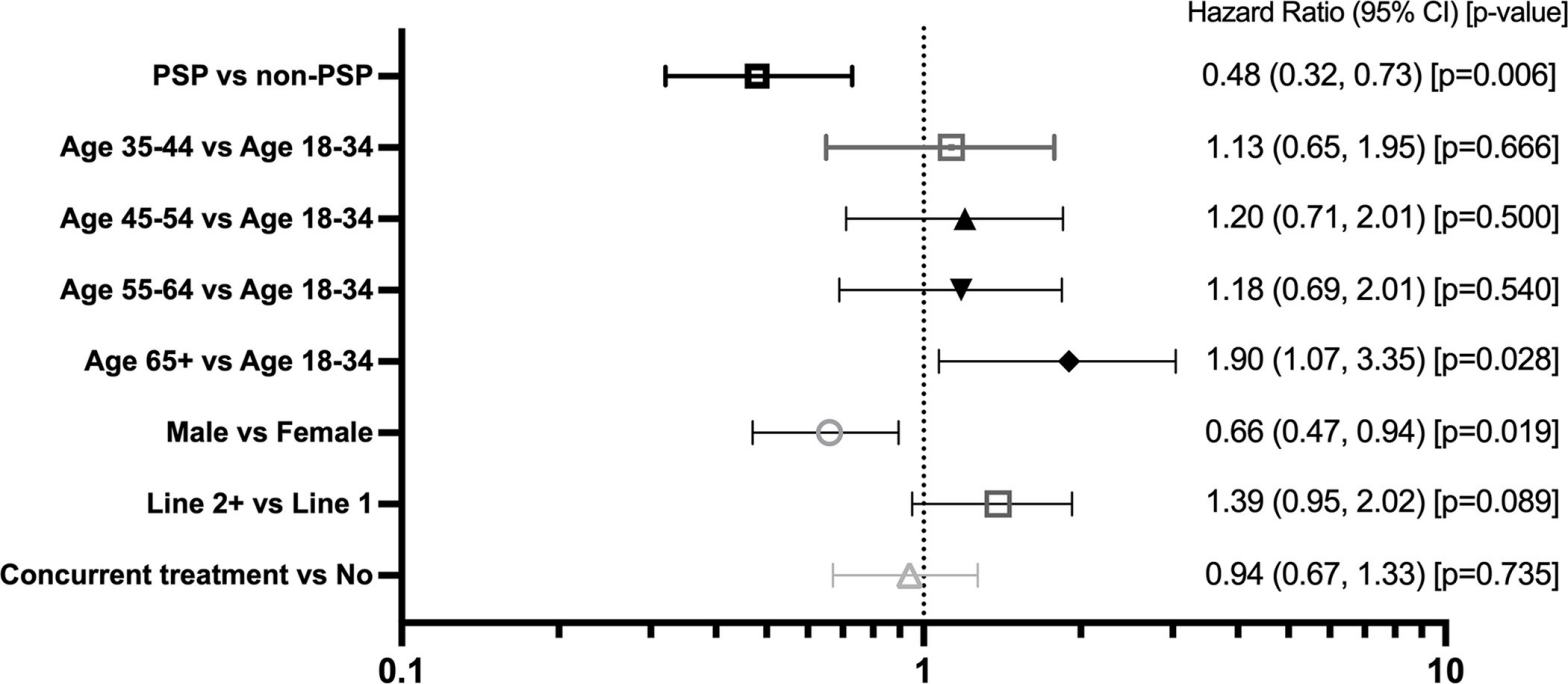
Hazard-Ratio (HR) for adalimumab discontinuation across all pooled indications using the Primary Analysis Sets from the prospective PSP and retrospective PBS 10% non-PSP cohorts. A Cox regression, weighted by the inverse probability of treatment weighting (IPTW), was used to calculate, across all pooled indications, the hazard-ratio (HR) comparing the hazard (risk) of adalimumab discontinuation in (i) the PSP cohort versus the PBS 10% non-PSP cohort; (ii) Patients in different age brackets versus aged 18–34; (iii) Male versus female patients; (iv) Later lines of bDMARD treatment versus first line bDMARD users; (v) patients on concurrent treatment versus no concurrent treatment.

In the final weighted and adjusted model for the comparison between the prospective PSP cohort and retrospective PBS 10% non-PSP cohort, patients enrolled in the PSP were less likely to discontinue compared to the non-PSP PBS 10% sample, as determined by log-rank test (p = 0.0007 (Fig 2B) and cox regression (HR [95% CI] = 0.44 [0.28 to 0.68], p<0.001) (Fig 3). Similar results were observed in the IPTW trimmed sensitivity analysis (HR [95% CI] = 0.51 [0.32 to 0.81], p<0.004; S1 Table) and in the sensitivity analysis using the broader IPTW adjusted PBS sample (HR [95% CI] = 0.44 [0.28 to 0.69], p<0.001, S1 Table). The persistence rate at 12 months in the IPTW adjusted model was 81% (95% CI 76%, 90%) in the PSP group and 61% (95% CI 56%, 67%) in the non-PSP PBS 10% sample (Fig 2B). As shown by the Cox regression analysis of the time to discontinuation (Fig 4), male patients were less likely to discontinue compared to female patients (HR 0.66, 95% CI 0.47 to 0.94, p = 0.0019), while patients over 65 years of age were also more likely to discontinue treatment compared to younger patients aged 18 to 34 years (HR 1.90, 95% CI 1.07, 3.35, p = 0.0028).

S2 Fig presents time to discontinuation Kaplan Meier curves in the RA and Rheumatology subgroups. Both subgroup analyses demonstrated significant difference in the survival functions of adalimumab time to discontinuation between the PSP and Non-PSP groups (Rheumatology Log-rank p = 0.001; RA Log-rank p = 0.008). The hazard ratio findings are also presented in S3 Fig (RA HR [95% CI] = 0.43 [0.23, 0.81]; Rheumatology HR [95% CI] = 0.53 [0.34, 0.84]. The sample size was deemed insufficient to run indication-specific analyses for AS, PsA, or CD.

The median time until first discontinuation could not be derived for the PSP or Non-PSP cohorts as the proportion of patients who reported discontinuation was less than 50%.

### Other analyses

The secondary objective of the study was to assess the potential impact of the PSP on PROMs, using the secondary endpoints of changes in scores over time for EQ-5D-5L, DLQI, BMQ:General, WPAI:General Health, PGDA, and BASDAI. This analysis was only possible in the prospectively enrolled cohorts as the PBS 10% database does not include such measures. The MMRM analyses across all indications combined found no effect of enrolment in the PSP on changes over time in any PROMs (S2 Table). MMRM analyses for DLQI scores could not be performed due to insufficient patient numbers. Independently of PSP status, all PROMs except BMQ Overuse, BMQ Harm (RA subgroup only), and WPAI Percent work time missed due to health, demonstrated statistically significant improvements in scores over time after adalimumab initiation, across all indications combined, as well as in the Rheumatology and RA subgroups (S3 Table).

Time to adalimumab discontinuation was also compared between the overall prospectively enrolled patients (FAS) who reported having received self-injection training and those who reported no-self-injection training using an unweighted Cox Regression. Across all indications, adalimumab discontinuation dates were reported by 23 (20%) patients who received self-injection training, compared with 11 (17%) patients who did not receive self-injection training. Self-injection training status was not significantly associated with adalimumab discontinuation, as determined by both log-rank test (p-value = 0.5577) and Cox regression (HR (95% CI): 1.239 (0.604, 2.542), p-value: 0.5587) (S4 Table).

## Discussion

This was a post-marketing observational study designed to assess the impact of a sponsor company-provided PSP on medication persistence for Australian patients initiating first-time PBS-reimbursed adalimumab for the treatment of several IMIDs. The majority of patients in any of the cohorts had been prescribed adalimumab for rheumatology conditions (RA, PsA or AS), rheumatoid arthritis being the most common diagnosis, and most were first-line bDMARD users.

Our results have shown that prospectively recruited patients who opted to enrol in the PSP were less likely to discontinue adalimumab treatment, and demonstrated higher rates of 12-month adalimumab persistence, compared to the retrospective non-PSP patients from the PBS 10% sample. Age and gender were also identified as factors influencing adalimumab discontinuation, with men less likely to discontinue than women and older patients (65+ years) more likely to discontinue than younger patients (aged 18–34 years). While the results from the retrospective comparison suggest a positive impact of the PSP on adalimumab persistence, no effect of PSP enrolment was shown on adalimumab discontinuation when comparing the two prospectively enrolled PSP and Non-PSP cohorts.

Several reasons might have contributed to these contrasting results. Firstly, there is no data to confirm that patients in the prospective non-PSP cohort didn’t subsequently enrol in the PSP at any time during the 12 months of follow up. Furthermore, the retrospective cohort can be considered a less biased non-PSP cohort, as the data sampling window was prior to the implementation of the first PSP for Australian patients prescribed adalimumab, meaning that these patients did not have the option to enrol on a PSP if support was needed but not provided by alternative sources. In contrast, enrolment into the prospective PSP or non-PSP cohort was based on intention to enrol in or decline the PSP. Although no data was collected regarding the potential utilisation of PSP-independent support services by prospectively enrolled patients in the study, it is reasonable to speculate that the patients who opted not to enrol in the sponsor company-provided PSP might have chosen so based on enough support already available to them through their treating physician and/or GP or other sources. Indeed, the high rate of adalimumab self-injection training received by the non-PSP cohort (49% of patients) suggests that some level of support was provided to at least half of the patients in this group. In further support of this hypothesis, in Australia some practices/hospitals have over time adopted standards of care including a nurse model that aims to build the capacity and capability of patient-centred communication and education. Where available, the nurse may undertake tasks such as coordinating and conducting assessments, scheduling reviews, and ensuring continuing of PBS funded therapy [28]. In addition to nurse support, specialised organisations across Australia, such as Arthritis Australia, are nowadays an important source of information and support services in the community, providing information on the disease and its management, self-management education programs, patient support groups and camps. These models of care, have been established in some Australian practices in relatively recent years. Another factor that may have confounded the results from the prospective cohorts, is that voluntary enrollment onto the study might have also enriched those cohorts with the most engaged patients, resulting in the prospective Non-PSP patients being more proactive in seeking the support needed from available sources and hence behaving more similarly to PSP patients. Furthermore, regular collection of PROMs that are not always routinely performed in clinical practice might have positively influenced patients’ outcomes in the prospective cohorts, possibly particularly affecting the non-PSP cohort, where the PSP, and hence additional touchpoints, were not to be provided.

All together these factors could at least partially explain the high adalimumab persistence rates observed in both PSP (78%) and Non-PSP (82%) prospective cohorts, as well as the absence of an observable impact of the PSP on adalimumab persistence when comparing these cohorts.

It should also be noted that the extent of uptake of PSP services (other than adalimumab self-injection training) by the patients enrolled in this study was not captured, hence it was not possible to stratify the results by level of PSP services utilisation. A supplementary analysis demonstrated no association between adalimumab discontinuation and nurse-driven self-injection training in the prospectively enrolled cohorts, independently of whether the patients were enrolled or not in the PSP. However, the proportion of patients who reported whether they received self-injection training, and hence included in this post-hoc analysis, was only 64% of the overall FAS (180/280). It is also worth noting that there are now both industry-provided and publicly available self-injection explanatory videos as well as in-services dedicated education support, and it is unknown whether these may have provided a comparable substitute for nurse driven self-injection training.

In agreement with the results of the retrospective component of this study, five comparable retrospective studies, have previously demonstrated an association between higher adalimumab persistence and PSP utilisation (S5 Table) [19, 21, 22, 29–31]. Differences in study setting and design (S5 Table) as well as discontinuation definition and PSP offerings and utilization might have contributed to the contrasting results found in our prospective comparisons. It should be also noted that published data demonstrate that a proportion of patients who initiate adalimumab will be unresponsive or lose response to treatment [16, 32, 33]. Depending on line of therapy, disease, and specific anti-TNF agent, the proportion of primary nonresponse can vary between 10–40% and secondary loss of response (early and beyond one year) between 5%-46% [14, 34–36]. Hence, the high adalimumab persistence rate observed in our prospective cohorts (78% in the PSP group and 82% in Non-PSP group) left little scope for further improvement. While comparably high adalimumab persistence rates have been reported in a Japanese population of RA patients [37], a previous retrospective study of real-world data from the Australian Optimising Patient outcome in Australian RheumatoLogy (OPAL) registry estimated a 12-month persistence rate of 54% among patients prescribed adalimumab for rheumatology indications between 2010 and 2016 [18]. Similarly low persistence rates were reported in two other retrospective studies analysing PBS 10% sample data from patients prescribed adalimumab for rheumatology indications, CD, or UC [38, 39]. Missing post-baseline data in our prospective cohorts is an important factor to consider when interpreting the contrasting persistence rates compared with the retrospective cohorts in our study and the previous studies. Reasons for study drop out were not collected and it is uncertain to what extent data was missing not at random. It is possible that some patients with missing post-baseline data could have discontinued adalimumab, leading to an underestimation of the discontinuation rate in our prospective population.

Some additional limitations of this study should be noted. The planned sample size for the two prospective cohorts was not reached due to lower than expected recruitment. The outcomes of the comparative analyses involving those two cohorts should therefore be interpreted with caution. In addition, assessment of a potential PSP effect based on indication was limited, as subgroup analyses other than RA and Pooled Rheumatology conditions could not be performed due to insufficient sample size. Also, the study population was most representative of patients with rheumatology diagnoses (RA, PsA or AS), who comprised over 70% of the Primary Analysis Set for both the prospective cohorts’ comparison and prospective PSP versus retrospective Non-PSP comparison. Rheumatoid arthritis was the most common diagnosis overall. In addition, most patients were first-line bDMARD users. Use of adalimumab as a second-line or more bDMARD was notably more common in the Non-PSP cohorts than the PSP cohort among patients with RA. However, the primary analyses were adjusted for line of therapy in the retrospective comparison, while line of therapy was not included in the final propensity score model for the prospective analyses given that the propensity score models including this variable were found to make negligible contribution to the overall model and analyses for the prospective comparisons. The strength of our retrospective cohort is that data extracted from the Services Australia 10% PBS dataset is considered nationally representative [24–27], and patients initiating adalimumab prior to any adalimumab PSP implementation (2009) can be considered a true non-PSP cohort. Indeed, the support needs of these patients couldn’t be addressed by a company-sponsored PSP and would have been less likely to be addressed by the alternative forms of support available nowadays. However, the non-contemporaneous nature of this historical cohort compared to the PSP cohort has also limitations. Despite the PBS eligibility criteria having remained the same for adalimumab treatment in IMID patients, it is likely that the early initiators of adalimumab using PBS subsidised treatment may differ from later initiators especially in terms of disease severity. To control for potential differences in the prospective and retrospective cohorts, the analyses were adjusted by age, sex, line of therapy and concomitant treatment in the IPTW weighted set, and a sensitivity analysis was run using a PBS 10% sample which included an additional year of sampling time compared to the primary sample. Another potential confounding factor when comparing two non-contemporaneous cohort of patients, is that the management of IMIDs might have changed over time. In particular, due to increasing understanding of the benefits of a treat-to-target approach in the management of IMIDs [40, 41], treatment might now be adjusted sooner in patients not responding optimally to treatment. However, this approach could potentially either increase or decrease medication persistence depending on whether treatment is optimized or switched, respectively. It could be argued that prior to the availability of more treatment options in recent years, there might have been a rationale for keeping patients on the same biologic even if response to treatment was less-than-optimal outcomes. If anything, this would have favoured persistence in the retrospective cohort.

It can be argued that overall these data support the efficacy of industry provided PSPs for biologic therapies used to treat chronic immune diseases. It can also be speculated that PSPs might have been more important for a wider proportion of patients in the early era of biologics, and/or more generally at the time of registration of any new medicine, as health care systems adapt to the required support models. Lastly, conclusions cannot be drawn as to impact of withdrawal of the industry PSP and if this would result in a drop in persistence in adalimumab patients, or whether the healthcare system would plug this gap. It is quite possible that HCPs/patients opting for the PSP are doing so due to gaps in support in their clinical centres.

## Conclusion

The results from the analyses of the prospective cohorts of this study indicate that irrespective of PSP utilisation, adalimumab persistence in this sample of Australian patients was high (79% overall at Month 12), with no significant difference between the PSP and non-PSP prospective cohorts over 12 months. In contrast, analyses comparing the prospective PSP cohort with a historical, retrospective cohort of patients from the PBS 10% sample who could not access a PSP show that patients enrolled in the company-provided PSP were less likely to discontinue treatment.

Together, the outcomes of both components of the study suggest that a structured industry-provided PSP is one of the factors in IMID patients’ management which has the potential to decrease the risk of adalimumab discontinuation, but only where persistence rates are suboptimal, potentially due to limited or lack of support services and/or education, which has become less common in contemporary periods of biologics use. Overall, this demonstrates that optimal adalimumab persistence can be achieved with either structured PSP or healthcare support from other sources. The findings may aid in identifying patients most likely to benefit from participation in a PSP.

## Supporting information

S1 Fig Adalimumab time to discontinuation Kaplan–Meier curves in rheumatology pooled indications and rheumatoid arthritis—Prospective PSP versus prospective non-PSP. Primary Analysis Sets. Analyses weighted by the inverse probability of treatment weighting (IPTW). S1A) Rheumatology pooled Indications (RA, AS, PsA). S1B) Rheumatoid Arthritis.
(TIF)

S2 Fig Adalimumab time to discontinuation Kaplan–Meier curves in rheumatology pooled and rheumatoid arthritis patients—Prospective PSP versus retrospective PBS 10% non-PSP. Primary Analysis Sets. Analyses weighted by the inverse probability of treatment weighting (IPTW). S2A) Rheumatology pooled Indications (RA, AS, PsA). S2B) Rheumatoid Arthritis.
(TIF)

S3 Fig Hazard-Ratio (HR) for adalimumab discontinuation in rheumatology pooled and rheumatoid arthritis patients. A cox regression, weighted by the inverse probability of treatment weighting (IPTW), was used to calculate the hazard-ratio (HR) comparing the hazard (risk) of drug discontinuation in the PSP cohort to the hazard (risk) in the non-PSP cohorts (either prospective or PBS 10% retrospective).
(TIF)

S1 Table Sensitivity analyses for adalimumab time to discontinuation Hazard-Ratio (HR). Prospective PSP versus Prospective non-PSP: (i) a sensitivity analysis was undertaken using propensity score trimming, where stabilised IPTWs below the 5th and above the 95th percentile were excluded. (ii) A second sensitivity analysis was undertaken whereby the weighting was omitted from the cox regression, allowing the analysis to include all patients in the FAS. Prospective PSP versus Prospective non-PSP: (iii) a sensitivity analysis was performed using a trimmed analysis set similarly to the prospective cohorts’ analyses, and (iv) a second sensitivity analysis was performed using a dataset which included an additional year of follow-up time for the non-PSP PBS 10% sample. A cox regression was used to calculate the hazard-ratio (HR) comparing the hazard (risk) of drug discontinuation in the PSP cohort to the hazard (risk) in the non-PSP cohorts (either prospective or PBS 10% retrospective). Kaplan–Meier log rank test p value is also reported.
(PDF)

S2 Table Weighted MMRM analyses for patient reported outcome measures (PROMs) in the prospective cohorts. Changes from baseline for PROM scores were analysed over time with a mixed model for repeated measurements (MMRM). MMRM model was also weighted by IPTW.
(PDF)

S3 Table Paired T-Test for change in PROMs over time in combined PSP and non-PSP prospective cohorts after starting adalimumab treatment (Primary Analysis Set). (PDF)

S4 Table Time to discontinuation by self-injection training status. Time to adalimumab discontinuation was compared between the prospectively enrolled patients who reported having received self-injection training and those who reported no self Injection training, using an unweighted Cox Regression. The Cox Regression model was implemented to calculate the adjusted hazard-ratio (HR), to compare the hazard (risk) of drug discontinuation in the Self-Injection Training cohort to the hazard (risk) in the No Self-Injection Training cohort.
(PDF)

S5 Table Comparison of studies investigating PSP impact on adalimumab persistence. (DOCX)
