# Supplementary figures and images for: Impact of a Patient Support Program on time to discontinuation of adalimumab in Australian adult patients with immune-mediated inflammatory diseases–an observational study
Source: PLoS One. 2024 Jun 13;19(6):e0300624. doi: 10.1371/journal.pone.0300624 (PMC11175455; doi:10.1371/journal.pone.0300624)

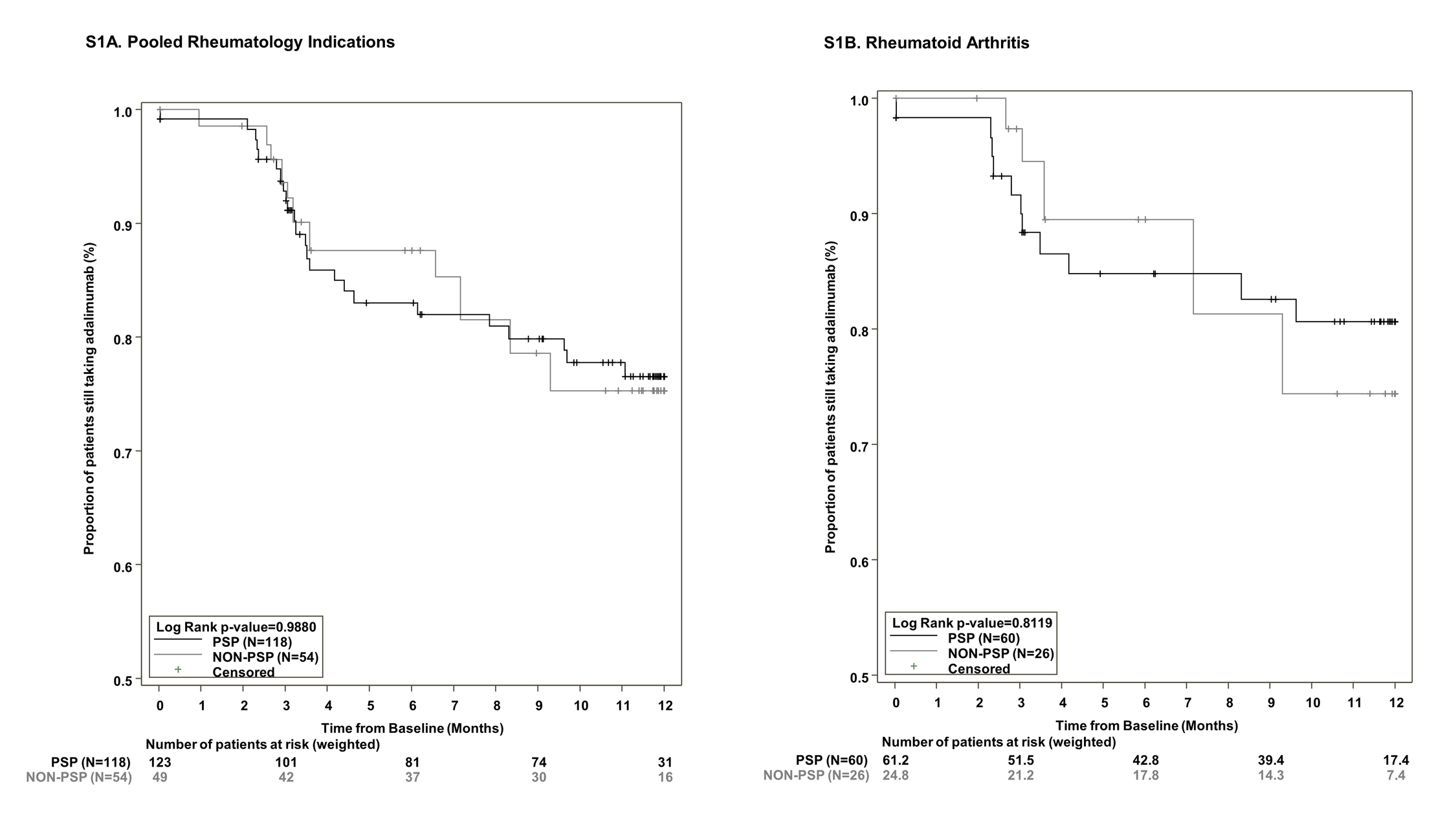

Supplement: S1 Fig — Primary Analysis Sets. Analyses weighted by the inverse probability of treatment weighting (IPTW). S1A) Rheumatology pooled Indications (RA, AS, PsA). S1B) Rheumatoid Arthritis. (TIF) [file pone.0300624.s001.tif]

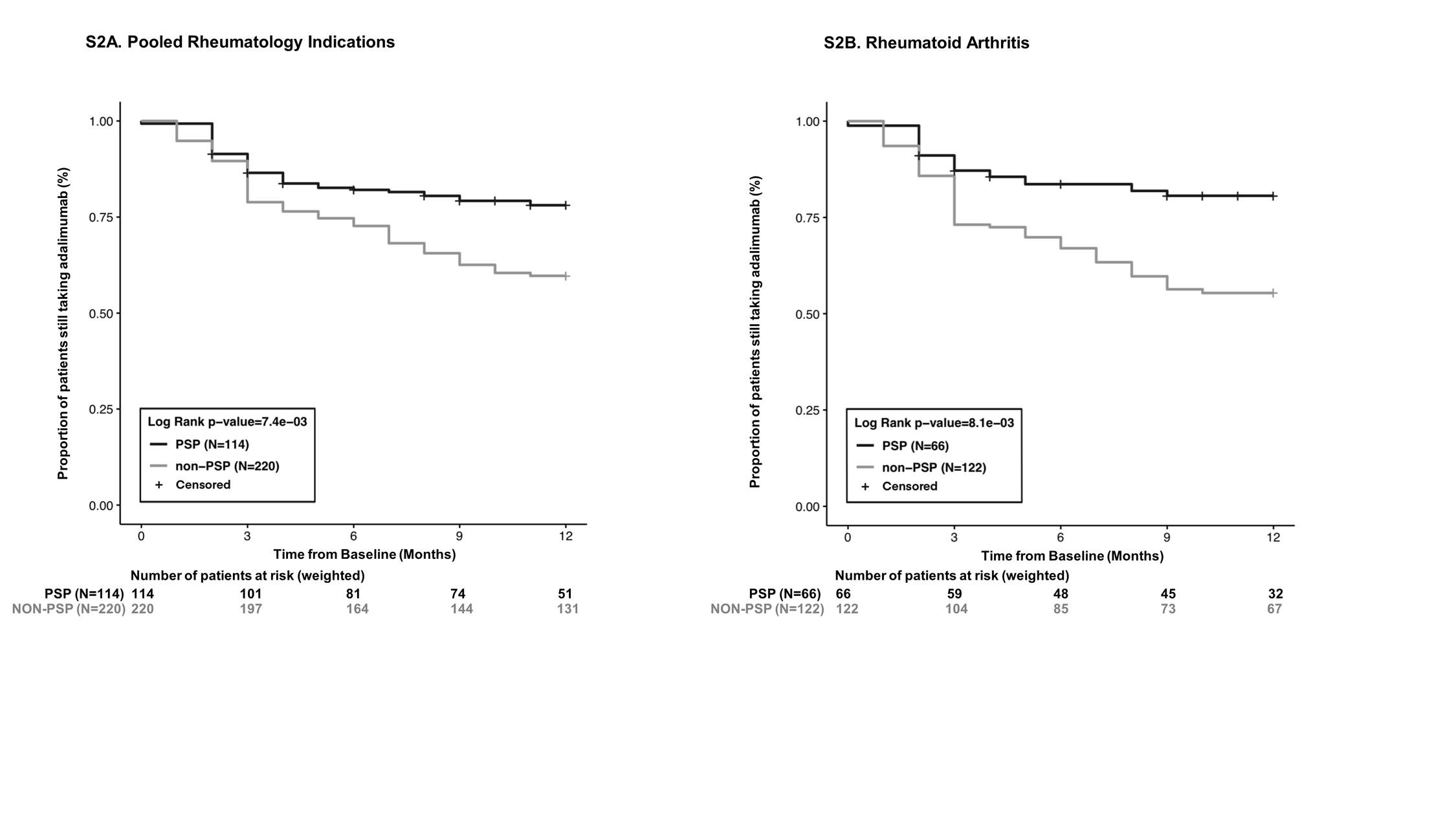

Supplement: S2 Fig — Primary Analysis Sets. Analyses weighted by the inverse probability of treatment weighting (IPTW). S2A) Rheumatology pooled Indications (RA, AS, PsA). S2B) Rheumatoid Arthritis. (TIF) [file pone.0300624.s002.tif]

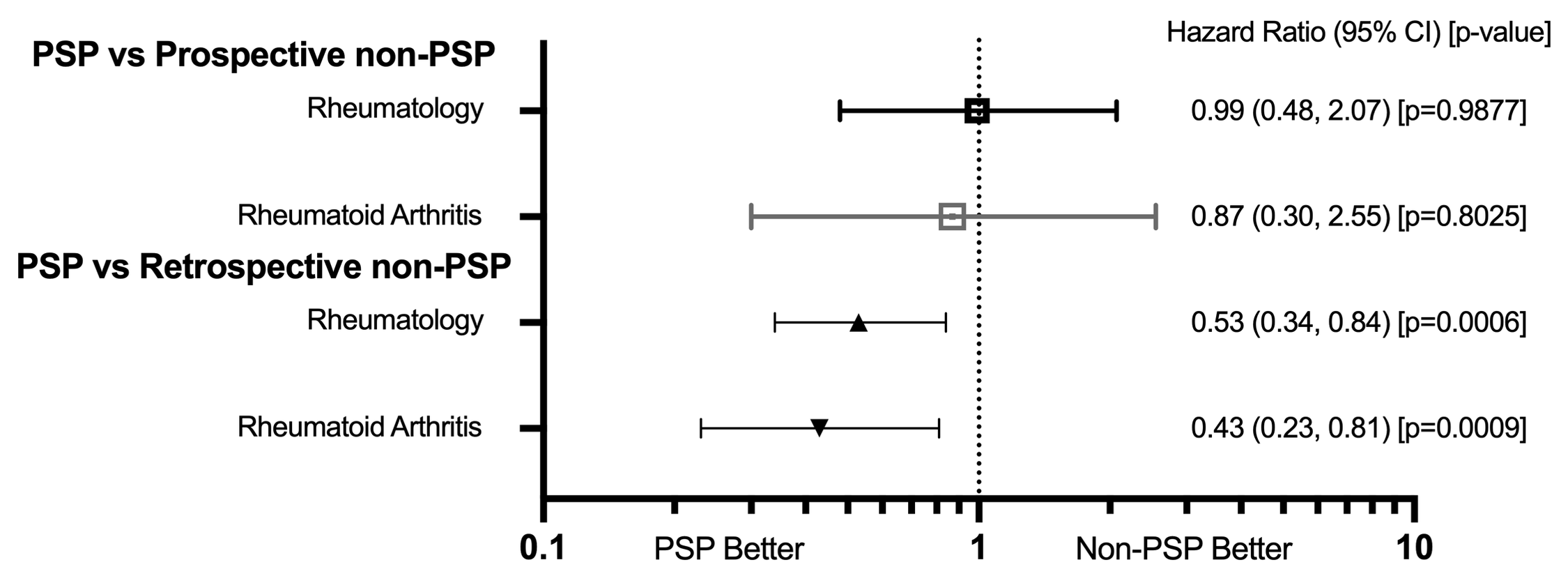

Supplement: S3 Fig — A cox regression, weighted by the inverse probability of treatment weighting (IPTW), was used to calculate the hazard-ratio (HR) comparing the hazard (risk) of drug discontinuation in the PSP cohort to the hazard (risk) in the non-PSP cohorts (either prospective or PBS 10% retrospective). (TIF) [file pone.0300624.s003.tif]
