# Supplementary material for: Impact of a Patient Support Program on time to discontinuation of adalimumab in Australian adult patients with immune-mediated inflammatory diseases–an observational study
Source: PLoS One. 2024 Jun 13;19(6):e0300624. doi: 10.1371/journal.pone.0300624 (PMC11175455; doi:10.1371/journal.pone.0300624)
Supplement: S1 Table — Prospective PSP versus Prospective non-PSP: (i) a sensitivity analysis was undertaken using propensity score trimming, where stabilised IPTWs below the 5th and above the 95th percentile were excluded. (ii) A second sensitivity analysis was undertaken whereby the weighting was omitted from the cox regression, allowing the analysis to include all patients in the FAS. Prospective PSP versus Prospective non-PSP: (iii) a sensitivity analysis was performed using a trimmed analysis set similarly to the prospective cohorts’ analyses, and (iv) a second sensitivity analysis was performed using a dataset which included an additional year of follow-up time for the non-PSP PBS 10% sample. A cox regression was used to calculate the hazard-ratio (HR) comparing the hazard (risk) of drug discontinuation in the PSP cohort to the hazard (risk) in the non-PSP cohorts (either prospective or PBS 10% retrospective). Kaplan–Meier log rank test p value is also reported. (PDF) [file pone.0300624.s004.pdf]

	Sensitivity Analysis	Weighted Log Rank Test<br>p-value	Weighted Cox Regression Analysis* Hazard Ratio (95% CI)	p-value
**PSP vs Prospective non-PSP**	(i) Trimmed†	0.4687	1.332 (0.654, 2.715)	0.4293
	(ii) Non-weighted¶	0.6283	1.186 (0.594, 2.366)	0.6289
**PSP vs PBS 10% Retrospective non-PSP**	(iii) Trimmed†	0.002642	0.51 (0.32, 0.81)	<0.004
	(iv) Broader PBS 10% sample#	< 0.001	0.44 (0.28, 0.69)	<0.001

CI = Confidence Interval

* Cox proportional hazards model includes PSP status as a factor

† A sensitivity analysis was undertaken using propensity score trimming, where stabilised IPTWs below the 5th and above the 95th percentile were excluded.

¶ A sensitivity analysis was undertaken where weighting was omitted from the cox regression, allowing the analysis to include all patients in the FAS.

A sensitivity analysis (IPTW adjusted) was performed using a dataset which included an additional year of follow-up time for the non-PSP PBS 10% sample
