# Supplementary material for: Impact of a Patient Support Program on time to discontinuation of adalimumab in Australian adult patients with immune-mediated inflammatory diseases–an observational study
Source: PLoS One. 2024 Jun 13;19(6):e0300624. doi: 10.1371/journal.pone.0300624 (PMC11175455; doi:10.1371/journal.pone.0300624)
Supplement: S2 Table — Changes from baseline for PROM scores were analysed over time with a mixed model for repeated measurements (MMRM). MMRM model was also weighted by IPTW. (PDF) [file pone.0300624.s005.pdf]

PROM	Time after starting adalimumab	p-value		
		All	Rheumatology	RA
EQ-5D-5L	PSP Status	0.4340	0.4173	0.2734
	Timepoint	0.5665	0.6939	0.4870
	PSP Status x Timepoint	0.7335	0.7126	0.2723
**BMQ Harm Score**	PSP Status	0.5190	0.4034	**0.0056**
	Timepoint	0.4375	0.3530	0.2029
	PSP Status x Timepoint	0.3255	0.6114	**0.0466**
**BMQ Overuse Score**	PSP Status	0.4691	0.6864	0.7015
	Timepoint	0.1037	0.4673	0.7194
	PSP Status x Timepoint	**0.0234**	0.7177	0.3115
**Percent activity impairment due to health**	PSP Status	0.4782	0.7548	0.6039
	Timepoint	0.2555	0.2042	0.4406
	PSP Status x Timepoint	0.9198	0.7930	0.8249
**Percent activity impairment while working due to health**	PSP Status	0.1885	0.3267	0.8776
	Timepoint	0.3144	0.3785	0.9984
	PSP Status x Timepoint	0.5802	0.5842	0.8868
**Percent work time missed due to health**	PSP Status	0.6225	0.9594	0.6697
	Timepoint	0.9307	0.6870	0.8206
	PSP Status x Timepoint	0.6193	0.2951	0.5173
**Percent overall work impairment due to health**	PSP Status	0.2001	0.3727	0.5968
	Timepoint	0.3403	0.6949	0.6327
	PSP Status x Timepoint	0.6134	0.9937	0.7198

	Time after starting adalimumab	p-value			
		Effect of Disease on well-being over last week		Effect of Disease on well-being over last six months	
		RA	RA+PsA	RA	RA+PsA
PGDA	PSP Status	0.9971	0.7395	0.0684	0.3674
	Timepoint	**<0.0001**	**<0.0001**	**<0.0001**	**<0.0001**
	PSP Status x Timepoint	0.4258	0.5080	0.6152	0.7662
