# Supplementary material for: Impact of a Patient Support Program on time to discontinuation of adalimumab in Australian adult patients with immune-mediated inflammatory diseases–an observational study
Source: PLoS One. 2024 Jun 13;19(6):e0300624. doi: 10.1371/journal.pone.0300624 (PMC11175455; doi:10.1371/journal.pone.0300624)
Supplement: S3 Table — (PDF) [file pone.0300624.s006.pdf]

	Time after starting adalimumab	p-value		
		All	Rheumatology	RA
EQ-5D-5L	Month 3	<0.0001	<0.0001	<0.0001
	Month 6	<0.0001	<0.0001	0.0019
	Month 12	<0.0001	<0.0001	0.0403
BMQ Harm	Month 3	0.2883	0.3712	0.2938
	Month 12	0.0449	0.0213	0.2272
BMQ Overuse	Month 3	0.2958	0.8691	1.0000
	Month 12	0.1225	0.5320	1.0000
Percent activity impairment due to health	Month 6	<0.0001	<0.0001	<0.0001
	Month 12	<0.0001	<0.0001	0.0002
Percent impairment while working due to health	Month 6	<0.0001	<0.0001	0.0127
	Month 12	0.0087	0.0015	0.0456
Percent work time missed due to health	Month 6	0.1827	0.0473	0.4841
	Month 12	0.6214	0.5169	0.6582
Percent overall work impairment due to health	Month 6	<0.0001	<0.0001	0.0320
	Month 12	0.0133	0.0014	0.0317

	Time after starting adalimumab	p-value			
		Effect of Disease on well-being over last week		Effect of Disease on well-being over last six months	
		RA	RA+PsA	RA	RA+PsA
PGDA	Week 1	0.0838	0.0759	0.0596	0.0360
	Week 2	0.5827	0.6229	0.3321	0.3480
	Week 3	0.8024	0.6445	0.2409	0.2183
	Week 4	0.5589	0.3138	0.6500	0.4543

	Week 8	0.1501	0.0813			0.8710	0.4263
	Month 3	0.0001	<0.0001			0.0045	0.0003
	Month 6	<0.0001	<0.0001			<0.0001	<0.0001
	Month 12	0.0034	<0.0001			<0.0001	<0.0001
