# Supplementary material for: Impact of a Patient Support Program on time to discontinuation of adalimumab in Australian adult patients with immune-mediated inflammatory diseases–an observational study
Source: PLoS One. 2024 Jun 13;19(6):e0300624. doi: 10.1371/journal.pone.0300624 (PMC11175455; doi:10.1371/journal.pone.0300624)
Supplement: S4 Table — Time to adalimumab discontinuation was compared between the prospectively enrolled patients who reported having received self-injection training and those who reported no self Injection training, using an unweighted Cox Regression. The Cox Regression model was implemented to calculate the adjusted hazard-ratio (HR), to compare the hazard (risk) of drug discontinuation in the Self-Injection Training cohort to the hazard (risk) in the No Self-Injection Training cohort. (PDF) [file pone.0300624.s007.pdf]

		Self Injection Training (n=120)	No Self Injection Training (n=65)
**Total Patients**	Total Patients	115	65
	Event (Total)	23 (20.0%)	11 (16.9%)
	Censored (Total)	92 (80%)	54 (83.1%)
**Log Rank Test**	p-value	0.5577	
**Cox Regression Analysis***	Hazard ratio (95% CI)	1.239 (0.604, 2.542)	
	p-value	0.55587	

CI: Confidence Interval Censored: Patients without the observed discontinuation event are censored at the last study contact date. *Cox proportional hazards model includes injection training status as a factor
