# Supplementary material for: Impact of a Patient Support Program on time to discontinuation of adalimumab in Australian adult patients with immune-mediated inflammatory diseases–an observational study
Source: PLoS One. 2024 Jun 13;19(6):e0300624. doi: 10.1371/journal.pone.0300624 (PMC11175455; doi:10.1371/journal.pone.0300624)
Supplement: S5 Table — (DOCX) [file pone.0300624.s008.docx]

| **Study** | **Setting** | **Patients^*^** | **Timeframe of adalimumab initiation** | **PSP** | **Persistence measure at 12 months follow-up** | | | |
| --- | --- | --- | --- | --- | --- | --- | --- | --- |
|  |  |  |  |  | Risk of discontinuation (hazard ratio [95% CI]) | Median time to discontinuation  PSP vs Non-PSP | Discontinuation rate  PSP vs Non-PSP | Persistence rate  PSP vs Non-PSP |
| **Prospective studies** | | | | | | | | |
| Current study (prospective cohorts only) | Australia | G, R, D | 2018-2019 | AbbVie Care | 1.219  [0.599-2.483]  *P* = 0.5851 | Unable to be derived |  | 78.0% vs 82% |
| **Retrospective Studies** | | | | | | | | |
| Brixner et al., 2019 [22] | United States | G, R, D | 2015-2017 | HUMIRA Complete with Nurse Ambassador |  | 350 vs 216.5 days  *P* < 0.0001 |  | 65.9% vs 51.5%  *P* < 0.0001 |
| Gerega et al., 2016 [30] | Canada | R | 2010-2014 | Care-coach calls | 0.282  *P* < 0.0001 |  |  |  |
| Rubin et al., 2017 [31] | United States | G, R | 2008-2014 | AbbVie PSP |  |  | 39.7% vs 46.2%  *P* = 0.001 |  |
| Srulovici et al., 2018 [32] | Israel | G, R, D | 2012-2014 | AbbVie PSP |  | 673 vs 574 days  *P* < 0.001 |  | 57.5% vs 45.6%  *P* < 0.001 |
| Fendrick et al., 2021 [19] | United States | G, R, D | 2015-2016 | HUMIRA Complete | 0.70  [0.64-0.78]  *P* < 0.0001 | 13.2 vs 8.4 months  *P* < 0.0001 |  | 49.0% vs 34.0%  *P* < 0.0001 |
| * Study population in terms of adalimumab indication, where G = gastroenterology, R = rheumatology, D = dermatology. | | | | | | | | |
